# Effect of Feed Additives Supplementation on the Growth Performance, Gastrointestinal Tract Characteristics, and Carcass Composition in Turkey Hens

**DOI:** 10.3390/ani12243464

**Published:** 2022-12-08

**Authors:** Alina Janocha, Anna Milczarek, Maciej Kosmalski, Paulina Gajownik-Mućka, Daniel Radzikowski

**Affiliations:** Institute of Animal Science and Fisheries, Faculty of Agrobioengineering and Animal Husbandry, Siedlce University of Natural Sciences and Humanities, Bolesława Prusa 14, 08-110 Siedlce, Poland

**Keywords:** microorganisms EM, Humokarbowit, garlic extract, nutrition, performance results, gastrointestinal tract, carcass value, turkey

## Abstract

**Simple Summary:**

Nowadays, in order to increase growth performance and carcass composition, alternative feed additives are sought after as a result of the withdrawal of antibiotics employed as growth promoters in poultry feeding. Studies on the simultaneous use of several feed additives in turkey diets are not currently available. Therefore, we conducted a study to see how effective three feed additives (effective microorganisms and Humokarbowit added to the feed and garlic extract added to the drinking water) included in the diets of turkey hens were on the growth performance, gastrointestinal tract characteristics, and carcass composition. It was shown that the included feed additives in the diets and water increased the final body weight and decreased the feed conversion ratio throughout the rearing period. Birds from the E group (fed with additives) scored a higher dressing percentage, and their muscularity and fattening were improved. The breast muscles of turkey from group E featured a lower pH_24_ and were of lighter colour compared with those of the birds from group C (fed without additives). The study revealed that for Big 6 turkey hens, the use of effective microorganisms and Humokarbowit in the birds’ diets and garlic extract in drinking water was recommendable in view of the improved growth performance and carcass composition.

**Abstract:**

In order to increase growth performance and carcass composition, including meat quality, as demanded by modern customers, alternative feed additives are sought after as a result of the withdrawal of antibiotics employed as growth promoters in poultry feeding. Therefore, we conducted a study to see how effective three feed additives added to the diets and water of turkey hens were. The experiment consisted of 200 Big 6 turkey hens divided into two equinumerous groups (C and E), with five subgroups in each. The 14-week-long growth performance study comprised five feeding periods. Both groups of birds were fed complete feed rations with mineral and vitamin supplements. The factor differentiating the groups were effective microorganisms and Humokarbowit added to the birds’ diets and garlic extract added to the drinking water in the experimental group (E) only. It was demonstrated that the included feed additives in the diets and water of turkey hens significantly increased (by 10%) the FBW and decreased (by 14%) the FCR throughout the rearing period. Birds from the E group scored significantly higher (by 3.6%) on the dressing percentage, and their muscularity and fattening grade were improved. Turkey fed rations containing the evaluated feed additives had a smaller share of the gastrointestinal tract in the body weight and a shorter duodenum and caecum (*p* ≤ 0.05). The muscles of turkey hens from group E featured a lower pH_24_ and were of lighter colour (*p* ≤ 0.05). To sum up, the use of effective microorganisms and Humokarbowit in the diets and garlic extract in the drinking water of turkey hens should be recommended in view of improved growth performance and carcass composition.

## 1. Introduction

Global poultry meat consumption has significantly increased within the past few decades. Turkey meat is the second most popular poultry meat worldwide. To efficiently meet consumer demands, intense genetic selection for rapid growth and higher meat yield has increased, resulting in increased body weight and breast meat proportions [1,2].

Environment-friendly feed additives constituting an alternative to already-withdrawn antibiotics that used to be added to poultry feeds or the chemotherapeutic agents now in use are being sought [3,4,5,6,7]. Among such additives, the most significant are probiotics, prebiotics, synbiotics, organic acids, herbs, and plant extracts [8,9,10,11,12]. 

Probiotics are mainly lactic acid bacteria that stimulate the development of the birds’ adaptive immunity to pathogenic bacteria. Although not a new concept, they have only recently begun to receive an increasing level of scientific interest [9,13,14,15]. The outcomes of studies [16,17,18] imply that poultry diets supplemented with adequate species of probiotic bacteria, on the one hand, prevent excessive growth of pathogenic microorganisms and, on the other hand, facilitate digestion processes and enhance the assimilability of nutrients, which consequently improves the productive and postslaughter performance of the birds. Milczarek et al. [18] showed that probiotic supplementation improved body weight and reduced the weight of the whole gastrointestinal tract in relation to the body weight of chickens. An improvement of the body weight and feed intake of broiler chickens fed a diet with probiotic was obtained by Rechman et al. [19]. Likewise, Awad et al. [20] demonstrated that the use of probiotics in broiler diet did not affect feed conversion ratio. According to the World Health Organization (WHO), Food and Agriculture Organization (FAO), and the European Food and Safety Authority (EFSA), selected probiotic microorganisms used in animal feeding should meet the basic general, functional, and technological requirements [21,22,23,24].

In recent years, in many countries (Germany, Australia, Great Britain, and Poland), an increase in the application of humic materials for animal feeding has been observed [25,26,27,28]. Humic substances are ubiquitous in natural and human-made environments such as soil, compost, sewage, natural waters, landfill leachates, and the atmosphere [29,30,31]. Piccolo [32] claimed that the vital humic materials’ role in maintaining environmental stability is generally accepted. Humic materials beneficially affect the growth of microorganisms. They also stimulate microbial growth, as a source of nutrients [33,34]. In addition, humic materials increase the solubility of poorly soluble substrates They enhance the survival and growth of microorganisms under unfavourable and adverse conditions due to antioxidant activity [35,36]. Kulikova et al. [29] and Olk et al. [31] formulated reasons that prevent the widespread use of humic materials in agriculture, namely, an insufficient number of field studies addressing the effects on humic products’ efficacy depending on environmental and management factors, a need for a mechanistic explanation of humic materials’ activity, a lack of quality control of humic products, and an insufficient number of long-term field trials. Humic preparations have been popular in ruminants [25], pigs [26], and poultry [27,28] rations. Humic additives generally have a positive influence on production results and on the health status of farm animals [25,26,27]. Islam et al. [37] claimed that humic substances added to diets can form complexes with metal ions, oxides, and clay minerals and can interact with organic compounds, e.g., fatty acids. Salejda and Krasnowska [26] showed that the addition of a humus-containing mineral preparation and rapeseed oil to pigs’ diet decreased cholesterol oxidation in pork. Eren et al. [28] noticed an improved egg production in the liquid humate group in the middle and late laying periods and the feed intake decreased in the early laying period while the feed conversion ratio improved in the middle laying period by supplementation of liquid humate. Dobrzański et al. [27] proved that the shell strength of eggs was significantly higher in hens fed with humic materials. The available literature lacks research results concerning the effectiveness of feeding turkey with humic additives. Nowadays, the interest of researchers [38,39,40,41] has also been focused on possible applications of garlic (*Allium sativum* L.) in various forms in poultry feeding. The chemistry of the Allium species is dominated by many sulphur-containing compounds. The major sulphur-containing compounds in intact garlic are γ-L-glutamyl-S-allyl-L-cysteines and S-allyl-L-cysteine sulphoxides (alliin) [42]. Garlic preparations and extracts have been shown to exhibit antiatherosclerotic, antimicrobial, hypolipidemic, antithrombotic, antihypertensive, and antidiabetic effects [43]. Dieumon et al. [39] demonstrated that garlic administered as an extract reduced the count of *Escherichia coli* and *Staphylococcus aureus* bacteria in the small intestine and the caecum of broiler chickens. Yasin et al. [44] claimed that that essential oil has less antimicrobial effect than garlic extract, and in higher concentrations, an inhibitory and bactericidal effect was observed for garlic essential oil. However, the antibacterial activity of garlic essential oil on Gram-positive bacteria (*Listeria innocua* and *Staphylococcus aureus*) was much higher than that of Gram-negative bacteria (*Escherichia coli* and *Pseudomonas aeruginosa*) [44]. Studies by Massad et al. [45] and Gbenda et al. [46] recorded a favourable effect of the garlic extract preparation on the birds’ weight gain and fattening grade. An improved weight gain and carcass quality and decreased mortality rates of slaughtered chickens after using a garlic preparation in the amount of 1–2.25 mL/kg feed were corroborated by Brzóska [40]. By contrast, Kim et al. [47] found that the use of garlic products in poultry diets improved the lipid profile and meat quality and, as a consequence, the texture and flavour of the meat. No influence of garlic or garlic and black cumin supplementation on the performance results, including relative organ weights as well as serum biochemistry and plasma, of broilers was obtained by Aydogan et al. [42].

Many authors [4,48,49,50] have showed that mixtures of various categories of feed additives used in animal nutrition are more effective since they ensure a good productive and postslaughter performance and a high quality of the meat, meeting the expectations of present-day consumers. However, the available references from the literature do not provide results regarding the use of mixtures of feed additives in turkey diets.

The present study was conducted in order to determine the impact of adding feed additives to the diets of turkey hens on their growth performance, gastrointestinal tract characteristics, and carcass composition.

## 2. Materials and Methods

### 2.1. Experiment Design

The experiment consisted of 200 Big 6 turkey hens divided into two equinumerous groups (C and E). Each group was additionally divided into five random replication subgroups. The number of birds in each subgroup was 20. Turkey hens were kept on litter in line with the intensive farming technology. Heating was provided by a central heating system and electric heaters (red light). Room temperature was set at 28 °C on the day of placement and was subsequently reduced by 2 °C per week. The temperature and humidity were recorded on a daily basis at 8 AM and 5 PM. Relative humidity was about 65 to 70%. Visual health inspection of all birds was performed on a daily basis. Turkeys had free access to the diets and water. The 14-week-long growth performance study comprised five feeding periods: 1–3 weeks, 4–6 weeks, 7–9 weeks, 10–11 weeks, and 12–14 weeks. Both groups of birds (C and E) received complete feed rations based on maize, wheat, soybean meal, soy oil, and lard (1:1), together with mineral and vitamin supplements. The nutritional value of the diets in the respective rearing periods (Table 1) matched the requirements of intensively growing birds [51]. The factor differentiating the groups was effective microorganisms (2.5 kg/t) and Humokarbowit (20 kg/t) added to the birds’ diets and garlic extract added to their drinking water (1.0 L/1000 L up to week 8 and 1.5 L /1000 L from week 9) in the experimental group (E) only. 

The EM used was grown for seven days in an adequate environment. In order to produce 25 litres of active EM preparation, 1 litre of SCD ProBio Original™ stock cultures and 1 litre of cane molasses, and 23 litres of water were used. The stock cultures of SCD ProBio Original™ included: *Bifidobacterium animalis, Bifidobacterium bifidum, Bifidobacterium longum, Lactobacillus acidophilus, Lactobacillus bulgaricus, Lactobacillus casei, Lactobacillus delbrueckii, Lactobacillus plantarum, Lactococcus diacetylactis, Lactococcus lactis* spp. *lactis, Streptococcus thermophilus, Bacillus subtilis* var. *natto, Saccharomyces cerevisiae, Rhodopseudomonas palustris,* and *Rhodopseudomonas sphaeroides.*

The Humokarbowit preparation used in the study had a multifaceted effect; it improved the digestibility and palatability of the feed, prevented diarrhoea, reduced the concentration of ammonium in farm buildings, and was a toxin binder. The preparation is patented in Poland and is composed of peat, humodetrinite, bentonite, and dolomite. Humokarbowit contains 30.1 g/kg crude protein, 135.5 g/kg crude fibre, 6.6 g/kg fat, and 209.7 g/kg crude ash, including 10–20 g/kg Fe, 50–100 mg/kg Zn, 25–50 mg/kg Cu, and 40–90 mg/kg Mn.

The water extract used contained a garlic extract, a mixture of flavouring substances, sodium chloride and magnesium sulphate. The alliin present in the extract is protective against oxidation, thanks to which the product maintains its biological activity. The manufacturer declares that the product does not damage the natural flora of the digestive tract of animals, therefore it can be used long-term and safely, without side effects. Alliin contained in the garlic extract lowers blood pressure, reducing the risk of the so-called sudden cardiac death in birds. During the growth performance study, the body weight of turkey hens was monitored individually at 1, 3, 6, 9, 11, and 14 weeks of life, along with the intake of feed per subgroup in respective rearing periods. These data were used for calculating the FCR.

$$\mathrm{FCR}\;\left(\frac{\mathrm{kg}}{\mathrm{kg}}\right)=\frac{\mathrm{feed}\;\mathrm{intake}\;\left(\mathrm{kg}\right)\;}{\mathrm{body}\;\mathrm{weight}\;\mathrm{gain}\;\left(\mathrm{kg}\right)}$$

The fattening efficiency was determined based on the European Production Index (EPI) using the formula:

$$\mathrm{EPI}=\frac{\mathrm{body}\;\mathrm{weight}\;\left(\mathrm{kg}\right)\times \;\mathrm{survivability}\;\left(\%\right)\times 100}{\;\mathrm{rearing}\;\mathrm{period}\;\left(\mathrm{days}\right)\times \;\mathrm{feed}\;\mathrm{conversion}\;\mathrm{ratio}\;\left(\mathrm{kg}/\mathrm{kg}\right)}$$

### 2.2. Postslaughter Characteristic Evaluation

On the last day of the feeding experiment, fifteen turkey hens representing the average body weight of each treatment were selected for the postslaughter assessment using the method of Ziołecki and Doruchowski [52]. After the birds had been eviscerated, the pH and length of respective gastrointestinal tract sections were measured, and the giblets (heart, liver, and gizzard) were weighed. 

The weights and lengths of the respective sections of the gastrointestinal tract were converted according to the preslaughter body weight of the turkey hens. The pH value of selected sections of the gastrointestinal tract of the turkeys was measured.

### 2.3. Physical Properties Evaluation of Muscles

Fifteen minutes after the slaughter the reaction (pH_1_) of their breast (m. pectoralis major) and thigh (m. iliotibialis) muscles was measured using a Testo 205 pH-meter with a dagger electrode. Next, the carcasses were cooled over 24 h at a temperature of 0–4 °C and afterwards, the reaction (pH_24_) of the muscles was measured again.

The colour of the pectoralis major and iliotibialis muscles was determined 24 h post mortem using a Minolta Chroma Metters (CR 300) instrument according to the L*, a*, b* system [53]. Two illuminant/observer combinations were applied, i.e., illuminant C (average day light) and standard observer 2_ as well as illuminant D65 (day light) and standard observer 10, recommended for measurements of meat colour [54]. In the used measuring system, L* denotes the psychometric colour saturation and is a spatial vector. On the other hand, a* and b* are trichromatic coordinates, where a* as a positive value corresponds to red, and as a negative value to green; in turn, a positive b* corresponds to yellow, and a negative b* to blue. The colour parameters a* and b* were used to calculate the chroma (C*_ab_) and the hue tone angle (*h*_ab_) with the formulas used by [55].

### 2.4. Statistical Analysis

A Student’s *t*-test for independent groups was used to compare means. Significantly different mean values, with a significance level of *p* ≤ 0.05, were marked with *. No * marking testified to the absence of statistically significant differences. The calculations were made using STATISTICA PL software, ver. 13.3 [56].

## 3. Results

The inclusion of effective microorganisms (EM) and Humokarbowit in the diets and garlic extract in the drinking water of turkey hens contributed to an increase (*p* ≤ 0.05) in body weight and a decrease (*p* ≤ 0.05) in feed conversion ratio (FCR) at respective rearing periods (Table 2). As a result, the final body weight of the turkey hens from group E was 10% higher, and concurrently, the FCR was lower by 14% compared with birds from group C. However, the feed intakes of the birds fed with additives were less (*p* ≤ 0.05) from 1 to 9 weeks, and next to the end of the rearing, they were bigger in comparison to those of the control group. In addition, a mixture of feed additives introduced into the diet for turkey hens reduced (by 10%) the survivability rate of birds, which, consequently, had a favourable effect on growth performance measured by EPI (*p* ≤ 0.05). 

Birds receiving feed rations containing the evaluated feed additives scored significantly (*p* ≤ 0.05) higher (by 3.6%) on the dressing percentage (Table 3).

Muscularity was slightly (*p* > 0.05) improved (53.31% vs. 52.64%), and, at the same time, the fattening grade measured as the percentage of skin with subcutaneous fat (7.61% vs. 8.71%; *p* > 0.05) and abdominal fat (0.29% vs. 0.39%; *p* ≤ 0.05) was smaller in birds from group E. No significant differences between the groups were recorded in the total percentage of giblets; however, the percentage of the liver was higher in birds fed diets with a mixture of feed additives.

The use of effective microorganisms and Humokarbowit in diets and garlic extract in the drinking water of turkey hens contributed to differences in the pH of respective sections of the gastrointestinal tract (Table 4).

A significant (*p* ≤ 0.05) increase in the concentration of hydrogen ions in the crops of birds from group E was found in comparison with turkey hens that did not receive any feed additives. In contrast, an opposite result was noted in the duodenum, caecum, and colon.

Turkey hens fed rations containing the evaluated feed additives (Table 5) had a significantly (*p* ≤ 0.05) smaller (by about 28%) share of the gastrointestinal tract in the body weight and a shorter duodenum (by about 20%) and caecum (by about 16%). 

The breast muscles of turkey hens receiving feed rations containing the evaluated feed additives had a lower pH_24_ (5.30 vs. 5.38; *p* ≤ 0.05) and were of lighter colour (L*) (47.72 vs. 45.10; *p* ≤ 0.05) than birds from group C (Table 6).

The type of diet had no influence on the physical traits (pH, colour) of the thigh muscles of turkey hens.

## 4. Discussion

Feeding practices should be focused on preserving the gastrointestinal tract’s microbial balance when chicken feed diets do not include growth boosters based on antibiotics [57]. Supplementing the feed rations for poultry with various feed additives allows one to achieve advantageous weight gains while reducing the individual feed conversion ratio, which was corroborated by our studies. However, it is difficult to discuss the results due to the lack of research regarding a similar composition of feed additives in poultry diets. Torres-Rodriguez et al. [58] and Lipiński et al. [59] demonstrated that a probiotic preparation (*Lactobacillus lactis*) introduced into feed rations for slaughter turkeys allowed to significantly increase (by 2.5–3.2%) their final body weight with no effect on the conversion of feed. The fact that *Bacillus cereus* var. *toyoi* included in turkey diets has no influence on productivity (final body weight and FCR) was reported by Biedrzycka et al. [60]. Stęczny and Kokoszyński [7] showed that Pro-Biotyk EM-15 and EMFarma probiotics caused an insignificant increase in body weight (42 days), feed intake, and feed conversion ratio (1–42 days) and an insignificant decrease in chicken mortality after four weeks of rearing. 

Krauze et al. [61] noted an increase in the final body weight of turkey hens and more efficient feed conversion after adding garlic extract to the drinking water. Similarly, Al-Shuwaili et al. [62] found that the final body weight of turkeys increased (*p* ≤ 0.05) and the feed conversion ratio decreased (*p* > 0.05) after the diet was enriched with 5% of garlic. In turn, the growth performance (weight gain, FCR) improved after introducing a mixture of garlic powder and *Lactobacillus casei* into the diet of broiler chickens in the study by Mangisah et al. [63]. In conclusion, the above-mentioned authors stated that the use of garlic powder and *Lactobacillus casei* could improve the nutrient digestibility and intestinal health, and as a result, the performance of broiler chickens. Many researchers [44,64,65] have claimed that essential oils and plant extracts, which are volatile and aromatic chemicals with good antibacterial activity, are among these components. However, the researchers have related the reason for this to differences in sex, species, place of plant growth, time of plant collection, soil type, and climatic conditions. Yasin et al. [44] showed that essential oil had less antimicrobial effect than garlic extract, and in higher concentrations, an inhibitory and bactericidal effect was observed for garlic essential oil.

The chicks used in the present investigations had acceptable health conditions. Since no invasive infections were found, the death rate of turkey hens was lower than that reported by Majewska et al. [38] and just higher than that calculated by Krauze et al. [61]. 

Stęczny and Kokoszyński [7] claimed that chickens exposed to probiotics (Pro-Biotyk EM-15 and EMFarma) did not differ significantly in terms of their dressing percentage and carcass composition. The carcasses of experimental chickens had a lower percentage of breast muscle, leg muscle, and abdominal fat, as well as a higher percentage of skin with subcutaneous fat compared with the carcasses of the control birds.

In analysing carcass composition, Mikulski et al. [4] did not find any effect of the herbal extract in turkey diets on the dressing percentage, muscularity, and fatness. A significant (*p* ≤ 0.05) increase in the dressing percentage and the share of giblets in the body weight coincided with the findings of Al-Shuwaili et al. [62]. They demonstrated that 5% of garlic included in the diet of turkeys increased their dressing percentage by 11.5% and the total share of giblets by 22.8%. These results were consistent with the findings of Langhout [66], who demonstrated that oil extracts could stimulate the digestive system of poultry, improve the function of the liver, and increase the activity of pancreatic digestive enzymes. The enhanced metabolism of oil, carbohydrates, and proteins in the major organs would increase the growth rate of those organs [67,68].

In analysing the weight of the gastrointestinal tract (GIT), expressed as a percentage of body weight, they noted a significant reduction in birds fed the experimental diet, which was consistent with the findings of Milczarek et al. [18]. In turn, Stęczny and Kokoszyński [69] demonstrated that at 42 days, Pro-Biotyk EM-15 and EMFarma™ supplemented broilers featured a significantly greater total intestine length and a higher intestine–body length ratio.

Similar to the study by Milczarek et al. [18], the pH in the duodenum and the caecum was higher in birds receiving probiotic diets. The pH was lower (*p* ≤ 0.05) in the crop and higher in the glandular stomach (*p* > 0.05) and the caecum (*p* ≤ 0.05), after the herbal extract was included in the feed rations of turkeys, which corroborated the results reported by Mikulski et al. [4]. Niba et al. [70] found that an increased acidity in the upper GIT was beneficial since it reduced or completely prevented the colonisation of the intestines by *E. coli* bacteria. 

Mangisah et al. [63] recorded a lower pH in the distal parts of the GIT (duodenum and caecum) in broiler chickens after introducing a mixture of garlic powder and *Lactobacillus casei* (GLC) into the birds’ diets. The GLC supplementation can decrease intestinal pH values. Inulin and FOS derived from garlic powder provide a specific substrate as a “nutrition source” that can be fermented by *Lactobacillus casei* and increases the growth of *Lactobacillus*, generally of lactic acid bacteria (LAB). An increased LAB growth correlates with an increased production of lactic acid and SCFA, which correlates with a decrease in intestinal pH. A high concentration of lactic acid decreases the pH and reduces the growth of harmful bacteria [71]. Many researchers [33,34,35,36] have claimed that humic materials beneficially affect the growth of microorganisms. In addition, humic materials enhance the survival and growth of microorganisms under unfavourable and adverse conditions due to antioxidant activity. Our obtained results on the pH in the distal parts of the GIT of turkey hens could be due to the interaction of the used additives.

Meat quality can be described based on its physical and nutritional attributes. The physical properties can be determined by the genetic line and nutrition [10,72,73]. Yalcin et al. [1] claimed that the selection for larger, faster-growing birds continues, and there has been a noticeable increase in meat quality defects. Defects such as pale, soft, and exudative (PSE) meat, and dark, firm, and dry (DFD) meat, affect the colour, water-holding capacity (WHC), myopathies, and texture of the product [74]. The evaluation of meat in terms of defects, if any, showed no relationship between its traits that would clearly allow us to confirm or exclude the presence of a PSE defect. In breast muscles, a lower pH was accompanied by a lighter colour of muscles, while it was the opposite in thigh muscles. The pH values in the breast muscles measured during the study were not in good agreement with those of Hiscock et al. [73]. By contrast, Owens et al. [75] found a PSE defect in turkey meat with pH = 5.72 and a lightness exceeding 54%. 

Pérez-Vendrell et al. [76] proved that natural xanthophyll pigments of plant origin were the main factor determining the colour of poultry products. Those authors demonstrated that the use of increasing amounts of supplements significantly increased the lightness of meat and altered the proportions between the remaining colour parameters. 

Islam et al. [37] and Salejda and Krasnowska [26] claimed that the addition of a humus-containing mineral preparation had a positive influence on the quality of animal raw materials.

## 5. Conclusions

This study leads to the conclusion that effective microorganisms EM (2.5 kg/t) and Humokarbowit (20 kg/t) should be recommended for use in the diets of Big 6 turkey hens, and garlic extract should be recommended for adding to the drinking water (1.0 L/1000 L up to week 8 and 1.5 L /1000 L from week 9) due to their improved growth performance and carcass composition. Moreover, existing research results on the assessed feed additives and their effectiveness in animal nutrition, as well as our results, encourage further research in this area.

## Figures and Tables

**Table 1:** Nutritive value of mixtures *.

Item	Rearing Period (Week)				
	1–3	4–6	7–9	10–11	12–14
ME (MJ/kg)	11.51	11.8	12.3	12.96	13.2
ME (kcal/kg)	2749	2817	2937	3095	3152
Crude protein (%)	27.0	25.0	22.5	20.1	19.6
Crude fibre (%)	3.3	3.4	3.5	3.5	3.1
Crude fat (%)	4.9	5.2	6.7	7.5	8.2
Lysine (%)	1.78	1.60	1.39	1.20	1.16
Methionine (%)	0.73	0.66	0.54	0.45	0.55
Crude ash (%)	7.1	6.7	5.7	4.9	4.4
Ca (%)	1.21	1.11	0.89	0.72	0.60
P available (%)	0.67	0.62	0.55	0.52	0.34
Na (%)	0.16	0.16	0.15	0.14	0.15
Mn (mg/kg)	140	140	114	108	86.40
Zn (mg/kg)	110	110	104.5	99	79.20
Fe (mg/kg)	80	80	47.5	45	36
Cu (mg/kg)	17.5	24.5	21.85	20.7	18
I (mg/kg)	3.0	3.0	1.90	1.8	1.44
Se (mg/kg)	0.35	0.35	0.29	0.27	0.22
Vitamin A (IU)	12,000	10,000	9500	9000	7200
Vitamin D_3_ (IU)	2500	2500	4512.5	4275	3420

* The mixtures contained: phytase (EC 3.1.3.26), antioxidants, and endo-1, 4- beta-ksylanase (EC 3.2.1.8). The experimental group rations contained more nutrients (from Humokarbowit), about 0.60 g/kg crude protein, 2.71 g/kg crude fibre, 0.13 g/kg fat, and 4.19 g/kg crude ash, and included 0.2–0.4 g/kg Fe, 1–2 mg/kg Zn, 0.5–1 mg/kg Cu, and 0.8–1.8 mg/kg Mn.

**Table 2:** Rearing results of turkey hens.

Item	Groups		SEM	*p*-Value
	C	E		
Body weight (kg)				
1 week	0.17	0.17	0.003	0.544
3 weeks	0.75	0.78	0.006	<0.05
6 weeks	2.45	2.55	0.017	<0.05
9 weeks	4.90	5.09	0.031	<0.05
11 weeks	6.78	7.05	0.045	<0.05
14 weeks	8.45	9.31	0.142	<0.05
Body weight gain (kg)				
0–3 weeks	0.58	0.61	0.005	<0.05
4–6 weeks	1.70	1.77	0.014	<0.05
7–9 weeks	2.45	2.54	0.015	<0.05
10–11 weeks	1.88	1.96	0.017	<0.05
12–14 weeks	1.67	2.26	0.099	<0.05
0–14 weeks	8.28	9.14	0.143	<0.05
Feed intake (kg)				
0–3 weeks	0.82	0.79	0.005	<0.05
4–6 weeks	4.29	3.47	0.137	<0.05
7–9 weeks	6.40	5.57	0.141	<0.05
10–11 weeks	6.18	6.37	0.051	0.052
12–14 weeks	4.78	5.07	0.051	<0.05
0–14 weeks	22.47	21.27	0.202	<0.05
FCR (kg/kg)				
0–3 weeks	1.41	1.31	0.018	<0.05
4–6 weeks	2.53	1.96	0.095	<0.05
7–9 weeks	2.61	2.19	0.069	<0.05
10–11 weeks	3.29	3.24	0.008	<0.05
12–14 weeks	2.86	2.24	0.140	<0.05
0–14 weeks	2.71	2.33	0.064	<0.05
EPI (points)	300	390	14.982	<0.05
Survivability (%)	92.3	95.4	0.250	<0.05

C—rations without additives; E—rations with effective microorganisms + Humokarbowit + garlic extract (in water); EPI—European Production Index; SEM—standard error of mean.

**Table 3:** Slaughter value of turkey hens.

Item	Groups		SEM	*p*-Value
	C	E		
Body weight before slaughter (g)	8.46	9.11	0.123	<0.05
Cold carcass weight (g)	6.73	7.51	0.070	<0.05
Dressing percentage (%)	79.56	82.47	0.543	<0.05
Share in cold carcass (%)				
Muscles total	52.64	53.31	0.317	0.315
including:				
Breast	29.58	30.14	0.346	0.456
Thigh	13.01	13.02	0.270	0.988
Drumstick	10.04	10.16	0.198	0.795
Abdominal fat	0.39	0.29	0.043	<0.05
Skin with subcutaneous fat	8.71	7.61	0.265	0.236
Share in body weight (%)				
Giblets total	2.43	2.58	0.253	0.406
including:				
Heart	0.32	0.33	0.009	0.777
Liver	1.01	1.24	0.052	<0.05
Gizzard	1.10	1.01	0.063	0.501

C—rations without additives; E—rations with effective microorganisms + Humokarbowit + garlic extract (in water); SEM—standard error of mean.

**Table 4:** pH value of selected sections of the gastrointestinal tract of turkeys.

Item	Groups		SEM	*p*-Value
	C	E		
Crop	4.93	4.21	0.129	<0.05
Glandular stomach	3.31	3.36	0.312	0.938
Duodenum	5.55	5.81	0.066	<0.05
Jejunum	5.49	5.95	0.174	0.212
Caecum	5.17	6.02	0.212	<0.05
Colon	4.49	5.88	0.244	<0.05

C—rations without additives; E—rations with effective microorganisms + Humokarbowit + garlic extract (in water); SEM—standard error of mean.

**Table 5:** Mass (g) digestive tract and length (cm) of selected segments, calculated for 1 kg of body weight.

Item	Groups		SEM	*p*-Value
	C	E		
Mass digestive tract	66.38	47.90	3.502	<0.05
Length of selected segments				
Duodenum	4.75	3.82	0.233	<0.05
Jejunum	26.66	24.22	0.741	0.100
Caecum	4.55	3.84	0.132	<0.05
Colon	1.62	1.70	0.052	0.518

C—rations without additives; E—rations with effective microorganisms + Humokarbowit + garlic extract (in water); SEM—standard error of mean.

**Table 6:** Physical properties of muscles.

Item	Groups		SEM	*p*-Value
	C	E		
Breast muscles				
pH_1_	6.21	6.02	0.099	0.353
pH_24_	5.38	5.30	0.016	<0.05
Colour				
L*	45.10	47.72	0.562	<0.05
a*	5.66	5.35	0.294	0.628
b*	−3.22	−2.95	0.356	0.735
C_ab_* = [(a*)^2^ + (b*)^2^]^0.5^	6.65	6.16	0.318	0.467
*h_ab_* = log (b*/a*)	−0.50	−0.51	0.054	0.969
Thigh muscles				
pH_1_	5.98	5.95	0.014	0.316
pH_24_	5.48	5.43	0.017	0.256
Colour				
L*	45.12	43.13	0.0861	0.270
a*	9.60	9.94	0.419	0.711
b*	−2.59	−2.57	0.444	0.978
C_ab_* = [(a*)^2^ + (b*)^2^]^0.5^	10.08	10.38	0.379	0.713
*h_ab_* = log (b*/a*)	−0.28	−0.26	0.045	0.833

C—rations without additives; E—rations with effective microorganisms + Humokarbowit + garlic extract (in water); L*—lightness, a*—redness, b*—yellowness, C_ab_*—chroma, *h_ab_*—hue tone angle; SEM—standard error of mean.

## Data Availability

The data are available on request from the corresponding author.
